# Performance of Particleboard Made of Agroforestry Residues Bonded with Thermosetting Adhesive Derived from Waste Styrofoam

**DOI:** 10.3390/polym16040543

**Published:** 2024-02-17

**Authors:** Tati Karliati, Muhammad Adly Rahandi Lubis, Rudi Dungani, Rijanti Rahaju Maulani, Anne Hadiyane, Alfi Rumidatul, Petar Antov, Viktor Savov, Seng Hua Lee

**Affiliations:** 1 School of Life Sciences and Technology, Institut Teknologi Bandung, Bandung 40132, Indonesia; rudi@sith.itb.ac.id (R.D.); rijanti@itb.ac.id (R.R.M.); anne@sith.itb.ac.id (A.H.); alfirumidatul@itb.ac.id (A.R.); 2 Research Center for Biomass and Bioproducts, National Research and Innovation Agency, Cibinong 16911, Indonesia; 3 Faculty of Forest Industry, University of Forestry, 1797 Sofia, Bulgaria; victor_savov@itu.bg; 4 Department of Wood Industry, Faculty of Applied Sciences, Universiti Teknologi MARA, Cawangan Pahang Kampus Jengka, Bandar Tun Razak 26400, Malaysia; leesenghua87@gmail.com

**Keywords:** adhesion, cohesion strength, maleic anhydride, MDI, particleboard, waste Styrofoam

## Abstract

This paper investigated the upcycling process of thermoplastic waste polystyrene (WPS) into thermosetting particleboard adhesive using two cross-linkers, namely methylene diphenyl diisocyanate (MDI) and maleic anhydride (MA). The WPS was dissolved in an organic co-solvent. The weight ratio of WPS/co-solvent was 1:9, and 10% of cross-linkers based on the WPS solids content were added subsequently at 60 °C under continuous stirring for 30 min. The adhesive properties, cohesion strength, and thermo-mechanical properties of WPS-based adhesives were examined to investigate the change of thermoplastic WPS to thermosetting adhesives. The bonding strength of WPS-based adhesives was evaluated in particleboard made of sengon (*Falcataria moluccana* (Miq.) Barneby & J.W. Grimes) wood and rice straw particles at different weight ratios according to the Japanese Industrial Standard (JIS) A 5908:2003. Rheology and Dynamic Mechanical Analysis revealed that modification with MDI and MA resulted in thermosetting properties in WPS-based adhesives by increasing the viscosity at a temperature above 72.7 °C and reaching the maximum storage modulus above 90.8 °C. WPS modified with MDI had a lower activation energy (*Ea*) value (83.4 kJ/mole) compared to the WPS modified with MA (150.8 kJ/mole), indicating the cross-linking with MDI was much faster compared with MA. Particleboard fabricated from 100% sengon wood particles bonded with WPS modified with MDI fulfilled the minimum requirement of JIS A 5908:2003 for interior applications.

## 1. Introduction

In the last five years, global plastics manufacturing has surpassed 360 million tonnes, with urban plastic waste management being one of today’s most serious environmental issues [1,2]. Among these polymers, expanded polystyrene (EPS) is defined as a low-density and low-cost polymeric material, which are compelling grounds for its widespread use in packaging, with a global yearly consumption rise of 6% and roughly 15 million tonnes produced globally. Styrofoam is a trade term that refers to a specific variant of EPS, a plastic material characterized by its lightweight and rigid nature. Due to its exceptional insulating qualities and cost-effectiveness, this material finds extensive utilization across several applications [1].

Regrettably, Styrofoam is not biodegradable and can last in the environment for extended periods, even after mechanical or photodegradation, hence representing a significant pollution hazard related to the accumulation of microplastics in the environment, causing harm to terrestrial and marine organisms [2]. In response to environmental considerations, many regions have implemented prohibitions or limitations on the utilization of Styrofoam containers and packaging, thereby promoting the adoption of alternative materials that are more ecologically friendly. Numerous biodegradable or compostable materials have the potential to function as substitutes for disposable Styrofoam products to mitigate their environmental repercussions. It takes around 500 years to decompose waste polystyrene (WPS) under optimal conditions [3]. In addition, polystyrene (PS) contains carcinogenic chemicals that leach into food and beverages. It can block drains, mainly affecting agriculture and fishing, and it takes up space in landfill sites [3,4]. Current methods of managing WPS include incineration, landfill, mechanical, chemical, and dissolution recycling. Globally, only 18% of WPS is recycled and 24% is incinerated [2,3,4]. Therefore, recycling WPS has potential benefits to the public and the environment.

Recently, products manufactured from recycled materials have gained increased research and industrial interest. The recent advances in recycling technologies of waste and by-products have resulted in optimized, economically feasible, and environmentally friendly value chains. The excessive amounts of disposed WPS worldwide represent a significant environmental hazard. Although direct recycling of Styrofoam into adhesives is not a common industrial practice, alternative methods exist for repurposing or reutilizing WPS. One of the prevalent techniques is the conversion of WPS into a substance called “recycled polystyrene”. The procedure entails the compression and fragmentation of WPS into smaller fragments, which can afterwards be employed as a primary constituent in diverse applications, such as the production of adhesives.

Wood adhesives represent a key element in the production of wood-based composites [5,6,7,8]. Currently, approximately 95% of wood adhesives used worldwide for manufacturing wood composites are based on formaldehyde. Urea–formaldehyde (UF) resins are the most widely used adhesives in wood-based panels, accounting for about 85% of the total volume worldwide, followed by melamine and phenolics [5]. The main drawback of these amino resins, besides their lower resistance in moisture conditions, limiting their application for interior purposes is the free formaldehyde emission from the finished panels, which is one of the main factors causing sick building syndrome. Several studies have reported methods to convert WPS into adhesives. Issam et al. prepared glue from WPS using coumarone–indene resin and benzene as tackifier and solvent, respectively [9]. The authors reported that the viscosity increased with polystyrene loading due to the concentration effect of polystyrene initially and entanglement at high polystyrene loading. It was found that the optimum concentration of polystyrene was 0.5 g/mL. Furthermore, Ahmetli et al. reported the chemical modification of industrial WPS with maleic anhydride (MA) in the presence of BF_3_·O(C_2_H_5_)_2_ [10]. The chemical modification was carried out in a single stage, without by-products, having high adhesion properties and resistance against aggressive sea conditions, which also makes it usable in practical applications.

Another study prepared wood–Styrofoam composite (WSC) panels that may be a very suitable solution for environmental pollution caused by WPS and also formaldehyde released from wood-based materials such as plywood [11,12]. WSC is manufactured by combining wood veneer sheets and Styrofoam. The mechanical properties of traditional plywood panels bonded with UF adhesive have been found to be higher than those of WSC panels. The usage of Styrofoam as an adhesive in the manufacturing of WSC panels causes a decrease in their mechanical properties. Therefore, the use of WPS directly as an adhesive is not recommended because thermoplastic Styrofoam melts at a certain elevated temperature [4].

The conversion of thermoplastic materials into a thermosetting polymer is achieved through a chemical process commonly referred to as cross-linking or curing [13,14]. This research work aimed to investigate and evaluate the feasibility of converting the thermoplastic behaviour of Styrofoam into a thermosetting adhesive as a way for upcycling WPS. For this purpose, the dissolution of waste Styrofoam in organic solvents and then cross-linking with polymeric isocyanate or maleic anhydride is proposed in the method. The adhesive’s basic properties, rheological properties, and thermo-mechanical properties were characterized to investigate the transformation of thermoplastic WPS into a thermosetting adhesive for particleboard manufacturing. Finally, the physical and mechanical performance of particleboard bonded with the WPS adhesives was evaluated by comparing with commercial UF adhesive.

## 2. Materials and Methods

### 2.1. Materials

Waste Styrofoam, technical grade toluene (95%), technical-grade butanol (99%), technical-grade propanol (99%), technical-grade methylene chloride (99%), technical-grade ethyl acetate (99%), and technical-grade butyl acetate (99%) were bought from local stores in Bogor, Indonesia. Methylene diphenyl diisocyanate (MDI, ±31% NCO content) was an industrial grade obtained from PT. Anugerah Raya Kencana, Tanggerang, Indonesia. Maleic anhydride (MA, 99%) was purchased from Merck, Darmstadt, Germany. Sengon (*Falcataria moluccana* (Miq.) Barneby & J.W. Grimes) wood particles were bought by PT. Sumber Graha Sejahtera, Serang, Indonesia, and rice straw was obtained from local paddy field in Bandung, Indonesia. Laboratory AquaDes was also used in this study. A low-molar-ratio UF adhesive at formaldehyde to urea mole ratio of 1.0 was prepared according to the published work [15].

### 2.2. Preparation of Waste Styrofoam-Based Adhesives

**Figure 1:**
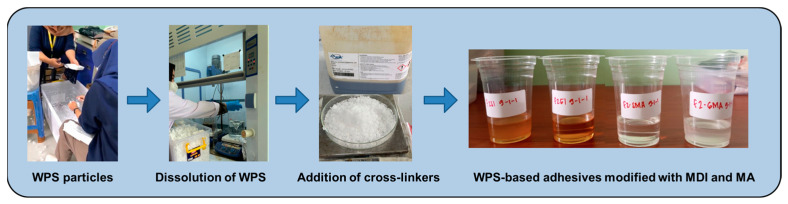
Preparation of the waste Styrofoam-based adhesives used in this work.

The waste Styrofoam used in this study was electronic containers (WPS-1) and food containers (WPS-2). Four types of WPS-based adhesives were prepared from WPS-1 and WPS-2 and then modified with MDI and MA (Figure 1). The WPS was cleaned with AquaDes and dried at room temperature. After that, the WPS was crushed into particles. The dissolution of WPS particles was adopted from a published work with some modifications [16]. Approximately 200 g (20% *w*/*v*) of WPS particles was dissolved in 1000 mL of co-solvents consisting of 100 mL of ethyl acetate, 100 mL pf methylene chloride, 150 mL of butyl acetate, 60 mL of propanol, 90 mL of butanol, and 500 mL of toluene. The dissolution process was performed at 60 °C under continuous stirring at 100 rpm for 10 min. After all the WPS particles were dissolved, around 40 g (20% *w*/*v*) of MDI based on the WPS solids was poured into the mixture. The cross-linking reaction was performed at 60 °C under continuous stirring at 100 rpm for 30 min. The modification with MA was performed following the above procedure.

### 2.3. Characterization of WPS-Based Adhesives

The basic properties of the WPS-based adhesives, such as solids content, viscosity, pH, and gelation time, were determined. The solids content of the WPS-based adhesives modified with MDI and MA was obtained by drying 1 g of the sample in an oven at 105 °C for 3 h and then dividing the oven-dried weight by the initial weight. The average viscosity of the WPS-based adhesives modified with MDI and MA was determined using a rotational rheometer (RheolabQC, AntonPaar, Graz, Austria) using a spindle no. 27 at 25 ± 2 °C and a constant shear rate of 100 s^−1^ for 120 s. The gelation time of WPS-based adhesives modified with MDI and MA was measured in boiling water at 10 rpm using a gelation time meter (GT-6, Techne Inc., Vernon Hills, IL, USA). The pH value of the WPS-based adhesives modified with MDI and MA was determined at 25 ± 2 °C using a digital pH meter (OrionStar A111, ThermoScientific, Waltham, MA, USA).

Fourier-transform infrared (FTIR) spectroscopy (SpectrumTwo, Perkin Elmer Inc., Waltham, MA, USA) was used to investigate the change in the functional groups of WPS, MDI, MA, and the WPS-based adhesives modified with MDI and MA. The samples were scanned at 25 ± 2 °C using the ATR method from 400 to 4000 cm^−1^ with a resolution of 4 cm^−1^ for each sample.

The rheological properties of the WPS-based adhesives modified with MDI and MA were investigated using a rotational rheometer (RheolabQC, AntonPaar, Austria). The dynamic viscosity, cohesion strength, and relaxation modulus of the adhesive were evaluated from 30 to 100 °C, under a constant shear rate of 200 s^–1^ and a heating rate of 5 °C min^–1^.

Dynamic Mechanical Analysis (DMA 8000, Perkin Elmer Inc., Waltham, MA, USA) was performed to investigate the thermo-mechanical properties of WPS-based adhesives modified with MDI and MA. Each adhesive was used to bond two Whatman filter papers, with a glue spread of 180 g m^–2^, to prepare a specimen with the dimensions of 50 mm × 8 mm × 0.2 mm. All specimens were pre-cured in an oven at 50 °C for 5 min before the DMA analysis. The storage modulus (E′), loss modulus (E″), and tan delta (tan δ) of each specimen were determined at a frequency of 1 Hz, strain level of 0.01%, and heating rate of 1, 3, and 5 °C min^–1^ in the scanning range of 30–200 °C in the dual cantilever mode.

The curing kinetics parameters were calculated using the Kissinger method [17]. Using the Kissinger method and data of the DMA, the curing kinetics parameters were obtained using the following equation.

$$\ln\left(\frac{\beta }{T_{p}^{2}}\right)=-\frac{E_{a}}{RT_{p}}+\ln\left(\frac{RA}{E_{a}}\right)$$

where *β* is the heating rate (K/s), *Tp* is the peak temperature (K), and *R* is the universal gas constant (8.314 J/mole K). The pre-exponential factor (*A*) was obtained from the intercept, and the activation energy (*Ea*) (kJ/mole) was calculated from the slope of the linear regression between ln (*β/Tp*^2^) and −1/*Tp*.

Morphological analysis was performed using a digital microscope (VHX 6000, Keyence, Osaka, Japan) at different magnifications with a VH-Z250T dual-light high-magnification zoom lens. Adhesive film was prepared by the polymer solution casting method. The evaporation of the solvents was performed in a fume hood at 25 ± 2 °C for 24 h.

### 2.4. Preparation of Particleboard

**Figure 2:**
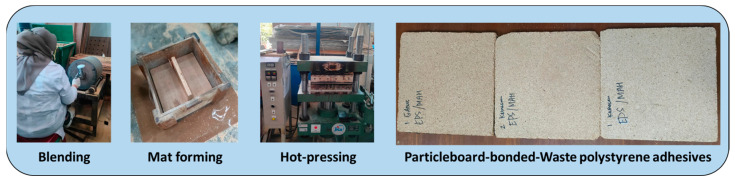
Preparation of particleboard bonded with WPS-based adhesive.

The preparation of a particleboard (PB) from agroforestry residues bonded with WPS-based adhesive is illustrated in Figure 2. The WPS-based adhesive was applied at the 30% level. The density of the PB was targeted at 0.8 g/cm^3^ with a panel size of 300 mm × 300 mm × 10 mm. The PB panels were fabricated at different mixtures of sengon wood particles and rice straw at a ratio (% wt/wt) of 100:0, 50:50, and 0:100, respectively. The calculated amount of particles was blended with the WPS-based adhesives, and the mixture was arranged in a wooden box for the mat-forming process. The mat was hot-pressed at a temperature of 180 °C for 30 min under 5 MPa of pressure using a compression moulding machine (Shinto, Kyoto, Japan). The PB panels were then conditioned at a temperature of 25 °C and relative humidity of 65% for 2 weeks.

### 2.5. Evaluation of Particleboard Properties

The properties of PB panels were evaluated according to the Japanese Industrial Standard (JIS) A 5908 (2003) for particleboard [18]. The parameters of physical properties were density, moisture content (MC), thickness swelling (TS), and water absorption (WA). The density and MC were measured with three replications for each formula. The WA and TS of the PB were determined after 24 h of immersion in water. In addition, the mechanical properties, i.e., modulus of elasticity (MOE), bending strength (MOR), and internal bond (IB) strength, were evaluated using a universal testing machine (AGS-I, Shimadzu, Kyoto, Japan) with a load cell of 10 kN at a cross-head speed of 2 mm/min for an IB strength test, and 10 mm/min for MOE and MOR tests.

### 2.6. Statistical Analysis

The mean values of PB properties were compared using analysis of variance (ANOVA), and Duncan’s multiple range test at a significance level of α = 0.05 was performed to determine the best combination of WPS-based adhesive content and agroforestry residue formulation on PB properties. Statistical analysis was performed using SPSS v. 21.0 software (SPSS Inc., Chicago, IL, USA).

## 3. Results

### 3.1. Basic Characteristics of WPS-Based Adhesives

**Figure 3:**
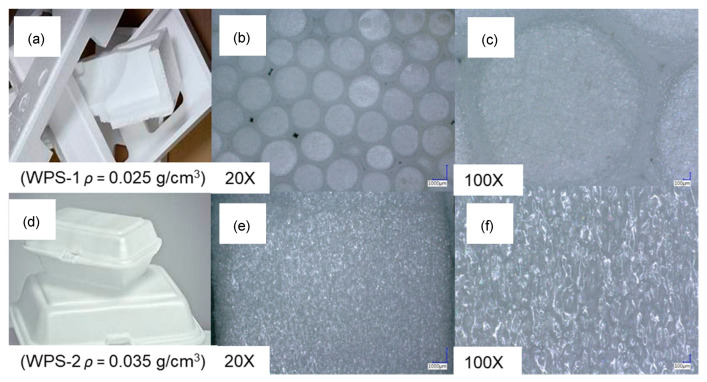
Digital microscope images of waste styrofoam. (**a**) WPS-1 at scale without magnification, (**b**) WPS-1 at 20 times magnification, (**c**) WPS-1 at 100 times magnification, (**d**) WPS-2 at scale without magnification, (**e**) WPS-2 at 20 times magnification, and (**f**) WPS-2 at 100 times magnification.

Styrofoam is a trade term for polystyrene (C_8_H_8_) which refers to a specific variant of expanded polystyrene (EPS), a plastic material that is light and stiff. In this study, an electronic container (WPS-1) and food container (WPS-2) were used as material for the plywood adhesive. Figure 3 shows that WPS-1 has a density of around 0.025 g/cm^3^, which is lower than WPS-2 (0.035 g/cm^3^), implying that WPS-2 has a denser structure than WPS-1. As depicted in Figure 3, the microscopy images of WPS-1 have a more porous structure compared to the WPS-2 and, therefore, lower density. The low density and porous structure of WPS-1 could produce more homogenous film formation of WPS-based adhesives [16].

To transform a thermoplastic material into a thermosetting polymer, the process of crosslinking must be initiated, forming a covalent linkage network among the polymer chains [19]. In this study, two cross-linkers, namely MDI and MA, were added to modify the properties of WPS-based adhesives. Table 1 presents the basic properties of the WPS-based adhesives modified with MDI and MA. The solids content of the WPS modified with MDI was higher than that modified with MA. The WPS modified with MDI had a range of solids content from 25.9% to 26.7%, while those modified with MA had a range of solids content from 12.4% to 13.6%. As a result, the WPS modified with MDI had a higher average viscosity (65.2–68.6 mPa·s) than the MA (24.8–26.7 mPa·s). There is a direct proportionality between solids content and viscosity. As the solids content increases, the viscosity of the adhesive tends to increase as well. This is because higher solids content often means a higher concentration of cross-linked polymers, leading to increased viscosity [20]. The gel time results followed a similar pattern to the solids content and viscosity. The time it takes for an adhesive to transition from a liquid to a gel state is referred to as gel time [21]. As the solids content increases, the concentration of cross-linked polymers increases, leading to increased viscosity and eventually shortening the gel time of the WPS-based adhesives. The WPS modified with MDI had a shorter gel time (6.4–8.6 min) compared to that modified with MA (10.2–13.5 min). The WPS-based adhesives, on the other hand, had lower solids content, viscosity, and gel time than UF adhesives. The pH of the WPS-based adhesives was categorized as acidic. Modification with MDI resulted in a pH of around 6.7–6.8, while the modification with MA resulted in a pH of around 2.5–2.6. The pH value obtained was lower than that of the UF adhesive, which had a pH value of 8.2.

### 3.2. Chemical Properties of WPS-Based Adhesives

**Figure 4:**
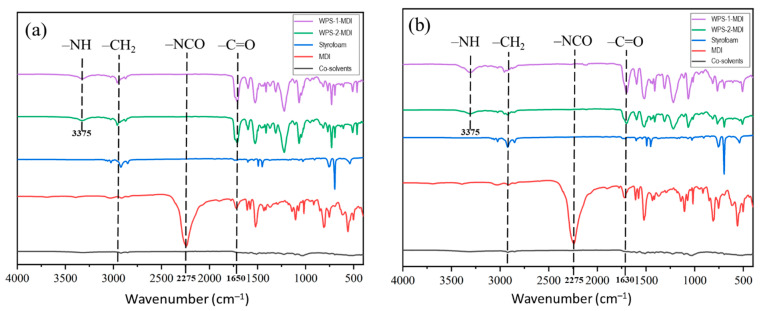
FTIR spectra of WPS-based adhesives modified with MDI. (**a**) Liquid adhesives and (**b**) cured adhesives.

**Figure 5:**
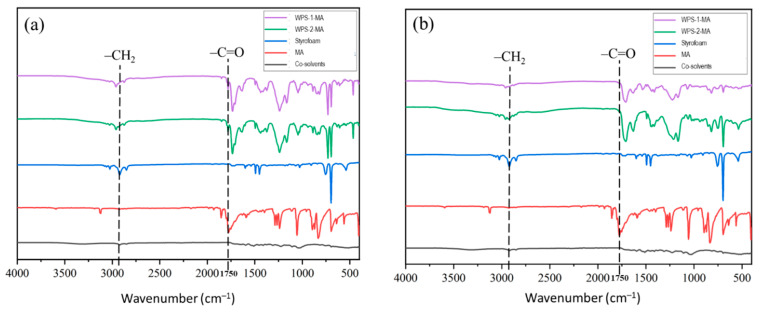
FTIR spectra of WPS-based adhesives modified with MA. (**a**) Liquid adhesives and (**b**) cured adhesives.

PS is a polymer composed of styrene monomers that have a styrene-based repeating polymer structure [1,2]. PS’s chemical characteristics are mostly determined by the functional groups contained in its structure. FTIR spectroscopy was applied to investigate the change in functional groups of the WPS-based adhesives modified with MDI and MA (Figure 4 and Figure 5). PS has stable aromatic benzene rings, methylene groups, vinyl groups, and benzyl groups. These functional groups could be involved in the cross-linking reaction of PS. The modification of WPS with MDI resulted in –NH and –C=O of urethane groups at wavenumbers 3375 cm^–1^ and 1650 cm^–1^, respectively. The urethane groups are formed based on the reaction of –N=C=O groups of MDI with the hydrogen atoms linked to carbon atoms in WPS [3,4]. There were no differences in the functional groups of the WPS-based adhesives modified with MDI in the liquid and solid state, indicating that the cross-linking reaction had occurred during the adhesive preparation in the liquid state. The modification of WPS with MDI resulted in –NH and –C=O of urethane groups at wavenumbers 3375 cm^–1^ and 1650 cm^–1^. The urethane groups are formed based on the reaction of –N=C=O groups of MDI at the wavenumber 2275 cm^–1^ with the hydrogen atoms linked to carbon atoms in WPS [3,4]. There were no differences in the functional groups of the WPS-based adhesives modified with MDI in the liquid and solid states, indicating that the cross-linking reaction had occurred during the adhesive preparation in the liquid state.

WPS modification with MA, on the other hand, resulted in different functional groups. Meanwhile, adding MA to the WPS-based adhesives produced the C=O groups with a wavenumber of 1750 cm^–1^ (Figure 5). This functional group reacted with the WPS to form C=O stretching vibration absorptions at 1031 cm^–1^, 1164 cm^–1^, and 1730 cm^–1^ for the ester group. This indicates that part of the anhydride opens the ring to form ester groups [22]. The presence of the ester groups could facilitate adhesion in bonding wood veneer. The absence of disparities in the functional groups of the WPS-based adhesives, whether in liquid or solid form, suggests that the cross-linking process took place during the adhesive synthesis in its liquid state.

**Figure 6:**
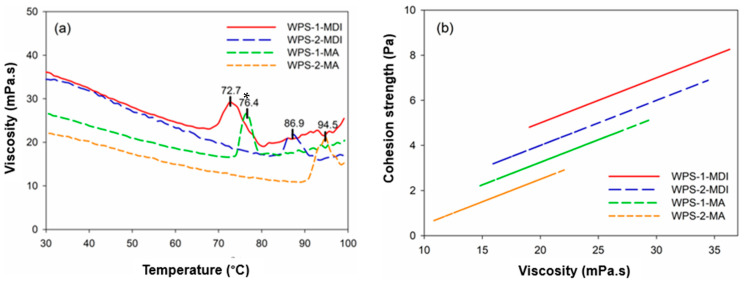
Dynamic viscosity of modified WPS-based adhesives as a function of temperature (**a**) and cohesion strength of modified WPS-based adhesives as a function of viscosity (**b**). Values with * refer to the peak temperature to reach a maximum viscosity of WPS-based adhesives.

The dynamic viscosity of the thermoplastic polymer is influenced by temperature, and the viscosity tends to decrease as temperature increases; this relationship is often non-linear [23,24]. Figure 6a shows that the viscosity of WPS-based adhesives decreased as the temperature increased, particularly from 30 °C to 65 °C, due to solvent evaporation. The modification of WPS with MDI increased viscosity to reach a maximum of 32.5 mPa·s at 72.7 °C for WPS-1 and 26.7 mPa·s at 86.9 °C for WPS-2. This indicated that the cross-linking process occurred above those temperatures and may change the thermoplastic to thermosetting behaviour. The viscosity might alter dramatically owing to the cross-linking. Initially, at high temperatures, the WPS-based adhesive may have a low viscosity, allowing it to flow and wet the surfaces to be attached. The dynamic viscosity tends to grow as the cross-linking reaction develops, suggesting the formation of the solid network structure [25]. By contrast, the modification of WPS with MA resulted in a different behaviour of dynamic viscosity than the WPS modified with MDI. The modification of WPS with MA resulted in an increase in viscosity, reaching a maximum of 28.6 mPa·s at 76.4 °C for WPS-1 and 25.8 mPa·s at 93.6 °C for WPS-2. The results showed that modification with MDI changed the thermoplastic behaviour of WPS into thermosetting, whereas modification with MA had no effect.

Adhesive viscosity and cohesion strength are typically directly related [20,21]. Adhesives with a higher viscosity have greater cohesion strength because their resistance to flow is linked to stronger molecular forces [16,26,27]. As shown in Figure 6b, the cohesion strength of WPS-based adhesives increased as the viscosity increased. When WPS-based adhesives were modified with MDI, the cohesion strength increased compared to WPS modified with MAs. As previously stated, the viscosity values of WPS modified with MDI were higher than those of WPS modified with MA, implying that the WPS modified with MDI has a stronger cohesion.

**Figure 7:**
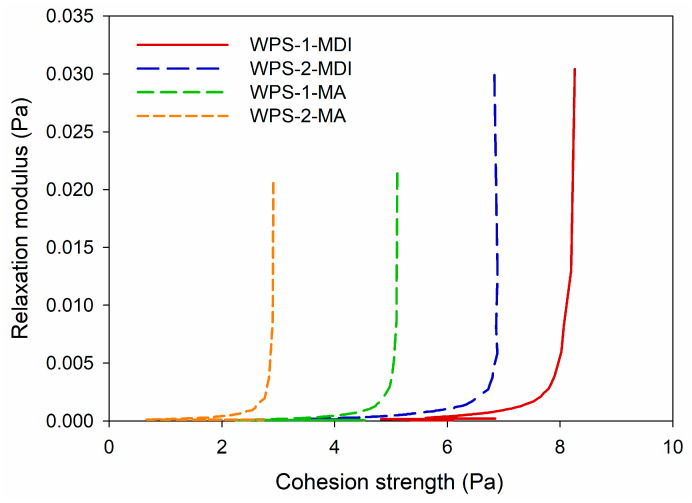
Relaxation modulus of modified WPS-based adhesives as a function of cohesion strength.

Cohesion strength and relaxation modulus are essential mechanical parameters of adhesives that provide information about how the material behaves under various loadings [16,28,29]. Figure 7 shows the relations between cohesion strength and relaxation modulus of WPS-based adhesives modified with MDI and MA. Cohesion strength refers to the adhesive’s internal strength or resistance to failure [16,30]. It assesses the adhesive’s capacity to hold together and resist internal fracture. Meanwhile, the relaxation modulus is a viscoelastic property that defines how a material responds over time to a constant tension or strain [31]. It measures a material’s ability to dissipate stress over time. WPS modified with MDI had a greater cohesion strength (5.4–8.3 Pa) and resulted in a higher relaxation modulus (0.028–0.031 Pa), while the WPS modified with MA had a lower cohesion strength (1.7–5.1 Pa) and relaxation modulus (0.019–0.022 Pa). This could be due to the higher viscosity of WPS modified with MDI than that of WPS modified with MA, suggesting the formation of the solid network structure from the cross-linking reaction. Higher cohesion strength and relaxation modulus could lead to better adhesion performance in wood bonding.

### 3.3. Thermo-Mechanical Properties of WPS-Based Adhesives

**Figure 8:**
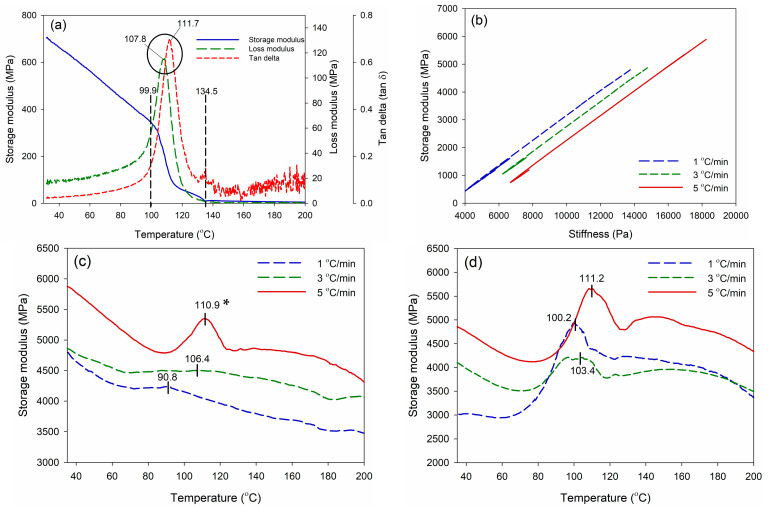
Thermo-mechanical properties of WPS-based adhesives. DMA thermogram of neat Styrofoam (**a**), storage modulus as a function of stiffness (**b**), storage modulus of WPS-MDI as a function of temperature (**c**), and storage modulus of WPS-MA as a function of temperature (**d**). Values with * refer to the peak temperature to reach a maximum storage modulus of WPS-based adhesives.

DMA is a method for determining the viscoelastic properties of materials, specifically adhesives [32]. DMA involves applying a dynamic oscillatory force or deformation to a sample and then measuring the resulting stress and strain responses. Figure 8 depicts the DMA thermograms of polystyrene and WPS-based adhesives modified with MDI and MA. Neat WPS without modification exhibited typical thermoplastic behaviour (Figure 8a). The storage modulus of neat WPS decreased as the temperature increased from 30 °C to 200 °C. The elastic component of a material’s response to an applied force is represented as the storage modulus. A higher storage modulus indicates a stiffer material [33], which could mean better load-bearing capacity and dimensional stability for an adhesive. Moreover, if the tan δ value is greater than 1.0, it signifies that the WPS-based adhesive is more viscous and has a capacity to disperse energy while undergoing deformation. A WPS-based adhesive with tan δ value below 1.0 signifies a greater elasticity, indicating that the material has a stronger inclination to retain energy. Modifying WPS with cross-linking agents like MDI and MA increased the storage modulus and stiffness (Figure 8b). DMA enables the detection of the peak temperature (Tp) of WPS-based adhesives at the maximum storage modulus (Figure 8c,d). A higher storage modulus may result in improved load-bearing capacity, increased dimensional stability, and a stiffer adhesive [34,35].

The *Tp* values at the maximum storage modulus are summarized in Table 2. The wPS-based adhesives modified with MDI and MA were scanned at different heating rates of 1, 3, and 5 °C/min. A higher heating rate generally results in a higher *Tp* value. WPS modified with MDI had lower *Tp* values (90.8–110.9 °C) than that modified with MA (100.2–111.2 °C). The *Tp* values and heating rates were used in the calculation of activation energy (*Ea*) according to the Kissinger method [36]. As a result, the WPS modified with MDI had a lower *Ea* value (83.4 kJ/mole) compared to the WPS modified with MA (150.8 kJ/mole). The *Ea* value is the energy barrier that must be overcome for a chemical reaction to occur. The *Ea* value is specifically linked to the kinetics of the curing or cross-linking events within the adhesive [37]. The lower *Tp* and *Ea* values indicated that the WPS modified with MDI could cross-link faster than the WPS modified with MA. The regression analysis of the Kissinger method showed a higher coefficient of determination (R^2^) value (0.9860) for the WPS modified with MDI compared to the WPS modified with MA (0.8075). The higher R^2^ value revealed that the WPS modified with MDI had a reliable Ea value according to the Kissinger method compared to the WPS modified with MA.

### 3.4. Morphological Characteristic of WPS-Based Adhesives

**Figure 9:**
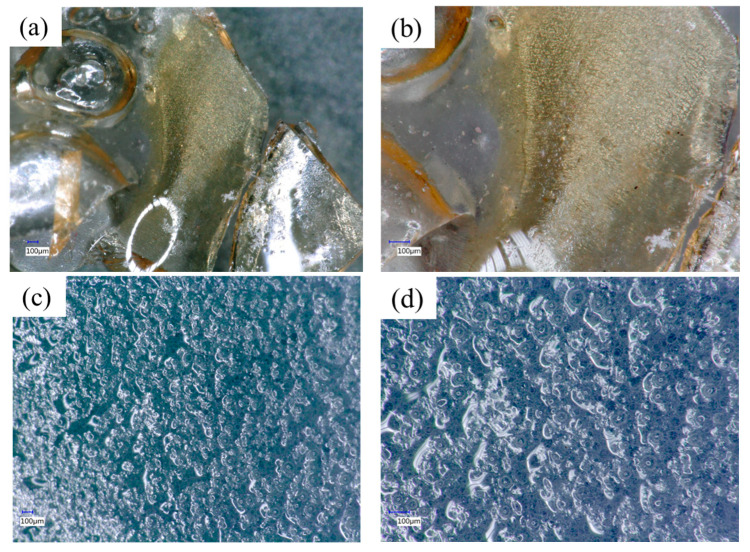
Morphological features of WPS-based adhesives captured by a digital microscope at different magnifications with dual-light high-magnification zoom lens. (**a**) 100× magnification of WPS modified with MDI, (**b**) 200× magnification of WPS modified with MDI, (**c**) 100× magnification of WPS modified with MA, and (**d**) 200× magnification of WPS modified with MA.

The morphological features of WPS-based adhesive film modified with MDI and MA are displayed in Figure 9. The WPS modified with MDI produced a smoother adhesive film owing to the cross-linking of MDI and WPS. The yellowish film of the WPS-based adhesive originated from MDI, as shown in Figure 9a,b. The WPS modified with MA, on the other hand, produced a rougher-surfaced adhesive film than the WPS modified with MDI (Figure 9c,d). The smoothness of the adhesive film can have a considerable impact on adhesion in bonding wood veneer [38]. Smoother adhesive films frequently result in greater adhesive–wood surface interaction by enhancing molecular interactions between the adhesive and wood [39]. As supported by the FTIR results (Figure 4 and Figure 5), the modification of WPS with MDI resulted in –NH and –C=O of urethane groups. The urethane groups are formed based on the reaction of –N=C=O groups of the MDI with the hydrogen atoms linked to carbon atoms in the WPS. In addition, the MA reacted with the WPS to form C=O stretching vibration for the ester group. This indicates that part of the anhydride of MA opens the ring to form ester groups. 

### 3.5. Evaluation of Particleboard Properties

Generally, the higher the average density of a particleboard panel, the greater its strength [40,41]. The average density of the particleboard panel was in the range of 0.70–0.74 g/cm^3^ (Table 3). The result was in the range of the target density, which was 0.7 g/cm^3^. The JIS A 5908:2003 standard requires a density of 0.4–0.9 g/cm^3^ for particleboard [18]. The MC of PB-manufactured agroforestry residues bonded with different contents of WPS-based adhesive is presented in Table 3. The results show that the MC of the particleboard was between 5.23% and 5.58%. The MC of the resulting PB panel was in the range of the JIS A 5908:2003 standard, which is 5.0–13.0%.

The thickness swelling of the particleboard made with different adhesives ranged from 30.5% to 53.7%. UF-bonded particleboard had the lowest thickness swelling of 30.5%, indicating that both WPS-based adhesives modified with MDI and MA had poorer water resistance than UF resin. However, all the particleboard produced in this study failed to meet the maximum allowable swelling limit of 12% as stipulated in JIS A 5908:2003. A similar trend was observed for water absorption as the UF-bonded particleboard had lower water absorption than the WPS-bonded particleboard. However, between modifications with MDI and MA, WPS-based adhesive modified with MDI had slightly superior water resistance than that of those modified with MA.

**Figure 10:**
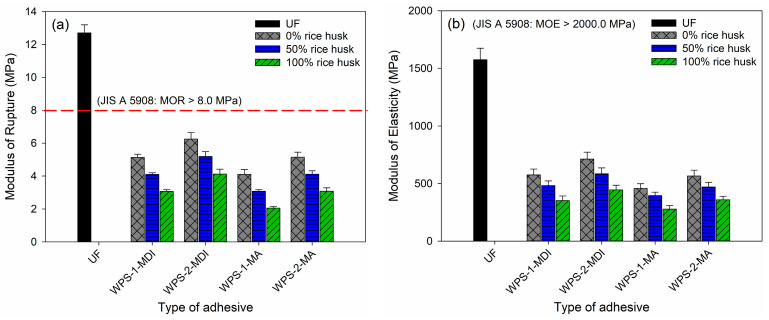
Mechanical properties of particleboard. (**a**) Modulus of rupture (MOR) and (**b**) modulus of elasticity (MOE).

**Figure 11:**
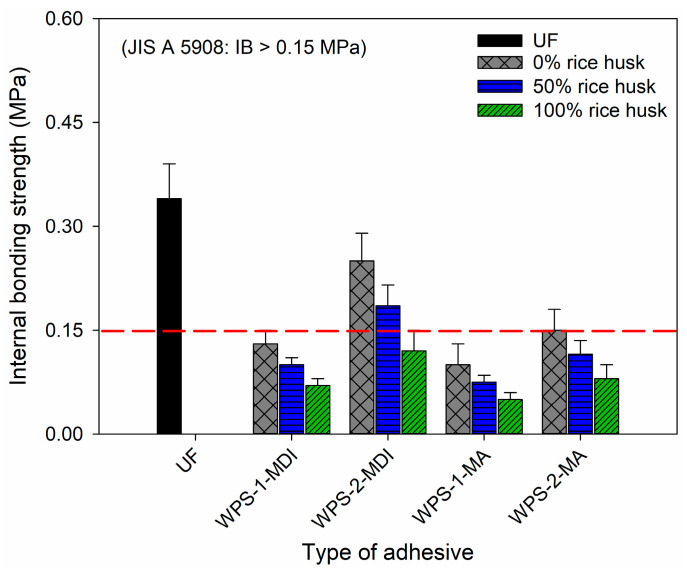
Internal bonding (IB) strength of particleboard.

Figure 10 and Figure 11 depict the modulus of rupture (MOR), modulus of elasticity (MOE) and internal bonding (IB) strength of the particleboard bonded with UF and WPS-based adhesives. It can be seen from the figures that those particleboards bonded with WPS-based adhesives had inferior MOR, MOE and IB compared to UF-bonded particleboard. All of the particleboards made with WPS-based adhesives failed to meet the minimum requirements of type-8 particleboard according to JIS A 5908. As for IB strength, only the particleboard made with WPS-1-MDI successfully met the minimum requirements of 0.15 MPa for type-8 particleboard. The addition of rice straws further hampered the mechanical performance of the particleboard. The higher the amount of rice straw added, the lower the mechanical properties of the particleboard [42,43].

## 4. Conclusions

In this study, thermosetting adhesive was successfully produced using waste polystyrene (WPS). WPS was used from two different sources, namely an electronic container (WPS-1) and a food container (WPS-2). During the adhesive preparation phase, the WPS was modified with MDI and MA. Overall, the results demonstrated that WPS modified with MDI had a higher solids content, viscosity, and shorter gel time than WPS modified with MA. It was discovered that the modification with MDI turned the adhesive into a thermoset, whereas MA did not affect the adhesion and cohesion properties of the adhesive. Furthermore, WPS-based adhesive modified with MDI has higher cohesion strength than MA. However, particleboard made with these adhesives performed poorly when compared to UF-bonded particleboard. The results showed that, despite having the potential to be a thermosetting adhesive derived from recycled resources, the adhesive’s mechanical properties were inferior to those of the UF resins. Particleboard fabricated from 100% sengon wood particles bonded with WPS modified with MDI fulfilled the minimum requirement of JIS A 5908:2003 for interior applications. More research is needed to improve the properties of WPS-based adhesives.

## Figures and Tables

**Table 1:** Basic properties of WPS-based adhesives modified with MDI and MA.

Adhesive Type	Solids Content (%)	Average Viscosity at 25 °C (mPa·s)	Gel Time at 100 °C (min)	pH
UF resins	58.4 ± 1.7	252.4 ± 5.1	3.5 ± 0.5	8.2 ± 0.3
WPS-1-MDI	25.9 ± 1.1	65.2 ± 2.3	6.4 ± 0.3	6.8 ± 0.2
WPS-2-MDI	26.7 ± 1.2	68.6 ± 3.8	8.6 ± 0.5	6.7 ± 0.2
WPS-1-MA	12.4 ± 1.2	24.8 ± 0.6	10.2 ± 0.4	2.6 ± 0.1
WPS-2-MA	13.6 ± 1.5	26.7 ± 0.4	13.5 ± 0.7	2.5 ± 0.1

**Table 2:** The activation energy (*Ea*) of WPS-based adhesives modified with MDI and MA.

Type of Adhesive	Heating Rates (°C/min)	*Tp* (°C)	*Ea* (kJ/mole)	R^2^
WPS-MDI	1	90.8	83.4	0.9860
	3	106.4		
	5	110.9		
WPS-MA	1	100.2	150.8	0.8075
	3	103.4		
	5	111.2		

**Table 3:** Density, moisture content, thickness swelling, and water absorption of particleboard bonded with WPS-based adhesives.

Adhesive Type	Particleboard Properties			
	Density (g/cm^3^)	Moisture Content (%)	Thickness Swelling (%)	Water Absorption (%)
UF	0.81 ± 0.02	5.23 ± 0.74	30.5 ± 0.51	65.7 ± 0.61
WPS-1-MDI	0.75 ± 0.02	5.32 ± 0.46	42.5 ± 0.55	73.2 ± 0.60
WPS-2-MDI	0.78 ± 0.02	5.36 ± 0.53	45.1 ± 0.45	77.5 ± 0.65
WPS-1-MA	0.70 ± 0.01	5.52 ± 0.56	51.5 ± 0.60	76.4 ± 0.66
WPS-2-MA	0.73 ± 0.01	5.58 ± 0.43	53.7 ± 0.75	79.3 ± 0.68

## Data Availability

The authors confirm that the data underlying the research are included in the article. The raw data that support the results are available upon reasonable request from the corresponding author.

## Abbreviations

Activation energy (*Ea*)	Moisture content (MC)
Attenuated total reflection (ATR)	Particleboard (PB)
Dynamic Mechanical Analysis (DMA)	Polystyrene (PS)
Expanded polystyrene (EPS)	Thickness swelling (TS)
Fourier-transform infrared (FTIR)	Urea–formaldehyde (UF)
Japanese Industrial Standard (JIS)	Waste polystyrene (WPS)
Internal bonding (IB)	Water absorption (WA)
Methylene diphenyl diisocyanate (MDI)	Weight by volume (*w*/*v*)
Maleic anhydride (MA)	Wood–Styrofoam composite (WSC)
Modulus of elasticity (MOE)	
Modulus of rupture (MOR)	
